# Supplementary material for: Spatiotemporal expression and control of haemoglobin in space
Source: Nat Commun. 2024 Jun 11;15:4927. doi: 10.1038/s41467-024-49289-8 (PMC11166948; doi:10.1038/s41467-024-49289-8)
Supplement: Supplementary file 2 — Reporting Summary [file 41467_2024_49289_MOESM2_ESM.pdf]

nature portfolio

Corresponding author(s): Christopher E. Mason, Joseph Borg, Afshin Beheshti

Last updated by author(s): May 30, 2024

Reporting Summary

Nature Portfolio wishes to improve the reproducibility of the work that we publish. This form provides structure for consistency and transparency in reporting. For further information on Nature Portfolio policies, see our Editorial Policies and the Editorial Policy Checklist.

Statistics

For all statistical analyses, confirm that the following items are present in the figure legend, table legend, main text, or Methods section.

n/a	Confirmed	
☐	☒	The exact sample size (*n*) for each experimental group/condition, given as a discrete number and unit of measurement
☐	☒	A statement on whether measurements were taken from distinct samples or whether the same sample was measured repeatedly
☐	☒	The statistical test(s) used AND whether they are one- or two-sided<br>*Only common tests should be described solely by name; describe more complex techniques in the Methods section.*
☐	☒	A description of all covariates tested
☐	☒	A description of any assumptions or corrections, such as tests of normality and adjustment for multiple comparisons
☐	☒	A full description of the statistical parameters including central tendency (e.g. means) or other basic estimates (e.g. regression coefficient) AND variation (e.g. standard deviation) or associated estimates of uncertainty (e.g. confidence intervals)
☐	☒	For null hypothesis testing, the test statistic (e.g. *F*, *t*, *r*) with confidence intervals, effect sizes, degrees of freedom and *P* value noted<br>*Give P values as exact values whenever suitable.*
☒	☐	For Bayesian analysis, information on the choice of priors and Markov chain Monte Carlo settings
☒	☐	For hierarchical and complex designs, identification of the appropriate level for tests and full reporting of outcomes
☒	☐	Estimates of effect sizes (e.g. Cohen's *d*, Pearson's *r*), indicating how they were calculated

Our web collection on statistics for biologists contains articles on many of the points above.

Software and code

Policy information about availability of computer code

Data collection: N/A

Data analysis:
BWA MEM (v0.7.15)
FastQC (v0.11.9)
MultiQC (v1.13)
Seurat (v4.2.0)
Cell Ranger (v6.1.1)
VGenes (vPre-release3)
pheatmap (v1.0)
fgsea (v1.22)
GSVA (v1.42.0)
bbtools (v38.92)
XTree (v0.92i)
Kraken2 (v2.1.2)
bracken (v2.6.2)
vegan (v2.6.2)
MetaSPAdes (v3.14.3)
MetaQUAST (v5.0.2)
Bowtie2 (v2.2.3)
samtools (v1.0, 1.9)
MMseqs2 (v13.4511)

Diamond (v2.0.14)
Bowtie2 (v2.4.1)
bedtools (v2.29.2)
R (v4.1.2)
Python3 (v3.10)
pheatmap (v1.0)
Seurat (v4.2.0)
Cell Ranger (v6.1.1)
DESeq2 (1.42.0)

For manuscripts utilizing custom algorithms or software that are central to the research but not yet described in published literature, software must be made available to editors and reviewers. We strongly encourage code deposition in a community repository (e.g. GitHub). See the Nature Portfolio guidelines for submitting code & software for further information.

Data

Policy information about availability of data

All manuscripts must include a data availability statement. This statement should provide the following information, where applicable:

- Accession codes, unique identifiers, or web links for publicly available datasets
- A description of any restrictions on data availability
- For clinical datasets or third party data, please ensure that the statement adheres to our policy

The JAXA CFE data is available via the NASA Open Science Data Repository's (OSDR)'s Biological Data Management Environment (https://osdr.nasa.gov/bio/) with accession numbers: OSD-5302, DOI: 10.26030/r2xr-h714. Deposited data from the sequencing data from the NASA Twin Study can be found on the NASA Life Sciences Data Archive (LSDA) and the accession code is not available due to privacy concerns. LSDA is the repository for all human and animal research data, including that associated with this study. LSDA has a public facing portal where data requests can be initiated (https://nlsp.nasa.gov/explore/lsdahome/datarequest). The LSDA team provides the appropriate processes, tools, and secure infrastructure for archival of experimental data and dissemination while complying with applicable rules, regulations, policies, and procedures governing the management and archival of sensitive data and information. The LSDA team enables data and information dissemination to the public or to authorized personnel either by providing public access to information or via an approved request process for information and data from the LSDA in accordance with NASA Human Research Program and JSC Institutional Review Board direction. The Inspiration4 data has been uploaded to two data repositories: the NASA Open Science Data Repository (osdr.nasa.gov; comprised of NASA GeneLab and the NASA Ames Life Sciences Data Archive (ALSDA)), and the TrialX database. Identifiers for publicly downloadable datasets in the OSDR are documented as follows: Data can be visualized online through the SOMA Browser (https://epigenetics.weill.cornell.edu/apps/I4_Multiome/), the single-cell browser (https://soma.weill.cornell.edu/apps/I4_Multiome/), and the microbiome browser (https://soma.weill.cornell.edu/apps/I4_Microbiome/). For the PBMC data, the data is available with OSDR accession ID: OSD-570 and the following link: https://osdr.nasa.gov/bio/repo/data/studies/OSD-570/. All source data used to produce the figures in this study are provided in the Source Data file.

Policy information about studies involving human research participants and Sex and Gender in Research.

Human research participants

Reporting on sex and gender	In the NASA Twins study, the sex of both twins is in the public domain. For the JAXA astronauts, this information was not made available for this study, while for the Inspiration4 crew, sex information was collected but all analyses were performed with the crew as a single cohort, irrespective of sex.
Population characteristics	Specific information for the JAXA astronauts was not available for this particular study. For the Inspiration 4 crew, the crew member composition was of two races and ages ranged from 29-51.
Recruitment	Participants for the Inspiration 4 were recruited by SpaceX and mission commander Jared Isaacman. Astronauts for NASA and JAXA are recruited by the specific agencies respectively.
Ethics oversight	All human astronauts subjects were consented at an informed consent briefing (ICB) at SpaceX (Hawthorne, CA), and samples were collected and processed under the approval of the Institutional Review Board (IRB) at Weill Cornell Medicine, under Protocol 21-05023569. All crew members have consented for data and sample sharing. JAXA and NASA astronaut data dissemination follows agency protocols.

Note that full information on the approval of the study protocol must also be provided in the manuscript.

Field-specific reporting

Please select the one below that is the best fit for your research. If you are not sure, read the appropriate sections before making your selection.

☒ Life sciences ☐ Behavioural & social sciences ☐ Ecological, evolutionary & environmental sciences

For a reference copy of the document with all sections, see nature.com/documents/nr-reporting-summary-flat.pdf

Life sciences study design

All studies must disclose on these points even when the disclosure is negative.

Sample size	For the the Inspiration4 crew, all crew members (n=4) were profiled, which was limited by the size of the Dragon capsule. 6 JAXA astronauts were considered, while the NASA Twin Study involved NASA's twin astronauts. Sample sizes were determined by availability of data from different missions
Data exclusions	No data has been excluded.
Replication	Replication tests are difficult as mission parameters cannot be repeated. Where possible, data validation was performed via western blots to validate proteomic findings.

Randomization	This is not relevant to the study as we were profiling the entire crew longitudinally (pre-flight, in-flight, and post-flight).
Blinding	Blinding was not possible because all subjects in the separate cohorts were astronauts in their respective crews.

Reporting for specific materials, systems and methods

We require information from authors about some types of materials, experimental systems and methods used in many studies. Here, indicate whether each material, system or method listed is relevant to your study. If you are not sure if a list item applies to your research, read the appropriate section before selecting a response.

Materials & experimental systems

n/a	Involved in the study
☒	☐ Antibodies
☒	☐ Eukaryotic cell lines
☒	☐ Palaeontology and archaeology
☒	☐ Animals and other organisms
☒	☐ Clinical data
☒	☐ Dual use research of concern

Methods

n/a	Involved in the study
☒	☐ ChIP-seq
☒	☐ Flow cytometry
☒	☐ MRI-based neuroimaging
